# Development and validation of the doctoral students’ time anxiety scale

**DOI:** 10.3389/fpsyg.2026.1710486

**Published:** 2026-05-14

**Authors:** Jianyue Chen, Jiajia Ren

**Affiliations:** 1 Academy of Civil Aviation Transportation, Chengdu Aeronautic Polytechnic University, Chengdu, China; 2 XuZhou Central Hospital, Xuzhou, China

**Keywords:** doctoral students, graduate education, grounded theory, scale development, time anxiety

## Abstract

Doctoral students represent a critical population for cultivating innovative talent, and their mental health directly impacts higher education quality and technological innovation capacity. However, the time anxiety commonly experienced by doctoral students in academic settings lacks systematic investigation, and no specialized time anxiety scale exists for this population. This study employed a sequential qualitative-quantitative mixed-methods approach, utilizing grounded theory to explore the structural dimensions of doctoral students’ time anxiety and empirical methods to develop a measurement scale. The results revealed that doctoral students’ time anxiety comprises three interconnected dimensions: affective anxiety, cognitive anxiety, and behavioral manifestations. These dimensions mutually influence one another, collectively shaping doctoral students’ temporal experiences and affecting their academic performance. The final 12-item Doctoral Students’ Time Anxiety Scale demonstrated satisfactory reliability and validity, making it suitable for measuring time anxiety and conducting empirical research among doctoral students. This scale provides a scientifically sound and effective tool for doctoral students’ mental health assessment, intervention design, and higher education management.

## 1 Introduction

With the acceleration of a new round of technological revolution and industrial transformation, doctoral education, as the highest level of knowledge production and talent cultivation, bears the crucial mission of cultivating high-level innovative talents in science and technology (Cardoso et al., 2022). However, beneath the noble academic mission and prestigious credentials, doctoral students often endure tremendous psychological burdens. Under the multiple pressures of high expectations, intense stress, and fierce competition, their mental health issues have become increasingly prominent, emerging as a significant concern worldwide. Recent studies consistently demonstrate that doctoral students face substantially higher mental health risks than the general population (Mackie and Bates, 2019; Estupiñá et al., 2024). A large-scale Belgian study in 2017 found that 32% of surveyed doctoral students suffered from severe mental illness, particularly depression, with over half experiencing suboptimal mental health (Levecque et al., 2017). This problem continues to intensify, as Nature’s global doctoral student tracking survey revealed that the proportion of doctoral students seeking external assistance due to stress during their studies increased from 36% in 2019 to 40% in 2022 (Woolston, 2022). In the Chinese context, this issue is even more severe: Among the 690 Chinese doctoral students participating in Nature’s 2019 survey, 40% sought help for depression and anxiety during their doctoral studies, significantly exceeding the global average (Woolston, 2019).

Among the various factors affecting doctoral students’ mental health, time anxiety emerges as a pervasive issue worthy of in-depth investigation. The acceleration trend in contemporary higher education and neoliberal management models has fundamentally reshaped doctoral students’ temporal experiences, subjecting them to unprecedented time pressures (Manathunga, 2019). Nature’s global graduate survey reveals that nearly 50% of doctoral students work more than 50 h per week, with 85% expressing dissatisfaction with their working hours (Woolston, 2019, 2022). However, doctoral students’ temporal predicament extends beyond mere working hours; the deeper issue lies in the fundamental conflict within their time experience. The modern academic environment creates a unique temporal paradox: While academic innovation inherently requires sufficient time for reflection and intellectual maturation, institutionalized academic evaluation systems often reduce innovative activities to quantified metrics measured against temporal scales (Vostal, 2015). This profound contradiction between external temporal constraints and internal academic needs causes doctoral students to oscillate constantly between “fast time” (task-oriented) and “slow time” (academic accumulation). Driven by meritocratic ideologies, doctoral students continuously invest time and chase deadlines in pursuit of academic excellence, paradoxically deepening their perception of time scarcity and anxiety (Sverdlik et al., 2018). This complex temporal experience interweaves tension, worry, and fear regarding the passage of time, generating a distinctive form of time anxiety. Therefore, time anxiety not only reflects the common predicament faced by doctoral students but also provides an important entry point for understanding and optimizing the doctoral education process.

Time anxiety, as a distinctive psychological phenomenon, has garnered attention across extensive psychological and sociological research. Zimbardo and Boyd (1999) Time Perspective Theory posits that individuals’ perception and interpretation of time profoundly influence their emotional experiences and behavioral decisions (Simons et al., 2004). Guzmán-Valenzuela and Barnett (2013), Shahjahan (2020), and Griffin (2022) further elaborate on how temporal experiences in academic environments shape researchers’ psychological states and work behaviors. While these theoretical perspectives provide important foundations for understanding doctoral students’ time anxiety, they have not yet formed a systematic conceptual framework specifically for this population. In terms of time anxiety measurement, the research trajectory demonstrates a progression from general to specific applications. Early measurement tools primarily originated from psychology, such as the Jenkins Activity Survey (JAS), which measures time anxiety related to Type A behavior patterns through dimensions including speed, impatience, and ambition (Boyd and Begley, 1987). Subsequently, researchers began exploring the multidimensional structure of time anxiety. Edwards et al. (1990) defined seven dimensions, namely, general speed, multitasking, rapid eating, rapid speech, impatience, punctuality, and time pressure. Conte et al. (1998) empirically condensed time urgency into six core dimensions: time awareness, time scheduling, decision performance, eating behavior, deadline control, and competitiveness. For broader temporal experience measurement, Macan et al. (1990) Time Management Behavior Scale (TMB) and Zimbardo and Boyd (2015) Zimbardo Time Perspective Inventory (ZTPI) provide measurement tools from the perspectives of time management behaviors and time orientation, respectively. Recent research trends toward developing more concise and practical measurement instruments, with work by Devoe and Pfeffer (2011) and Rudd et al. (2012) providing new pathways for rapid assessment of time anxiety. Despite the continuous enrichment of time anxiety measurement tools, existing research exhibits significant limitations. First, most measurement instruments are designed for general populations or workplace professionals, failing to adequately consider the specificities of academic environments. Second, existing tools predominantly focus on time management behaviors or time urgency rather than comprehensively capturing the multidimensional nature of time anxiety. Third, cross-cultural applicability issues limit the application of these tools across different educational systems. Most importantly, no measurement instrument currently exists specifically for doctoral students’ time anxiety experiences, a gap that severely constrains in-depth understanding and effective intervention of this issue.

Based on this foundation, this study aims to develop and validate a time anxiety scale specifically designed for doctoral students. The research employs an exploratory sequential mixed-methods design encompassing three consecutive phases: (1) qualitative research based on grounded theory to explore the structural dimensions of doctoral students’ time anxiety; (2) initial scale development and pilot testing, including item generation, content validity assessment, pilot testing, and item analysis; (3) formal scale construction and validation through exploratory factor analysis and confirmatory factor analysis to examine the scale’s reliability and validity. Unlike traditional scale development approaches that primarily rely on deductive methods based on *a priori* theoretical frameworks, this study adopts a mixed strategy emphasizing “bottom-up” induction supplemented by “top-down” deduction. Starting from doctoral students’ authentic experiences, the research deeply explores the essential structure of doctoral students’ time anxiety while drawing upon existing related research to refine measurement content. This approach ensures that the scale possesses both a solid theoretical foundation and alignment with the actual circumstances of doctoral students, achieving an organic integration of ecological validity and psychometric properties.

The research holds the following significance: At the theoretical level, it will enrich the interdisciplinary knowledge between temporal psychology and doctoral education research, providing new conceptual frameworks and measurement tools for understanding temporal experiences in academic environments. The structure of doctoral students’ time anxiety derived through grounded theory methodology will provide an empirical foundation for the development and validation of related theories. At the practical level, this scale can serve as an important tool for assessing doctoral students’ mental health, helping to identify individuals at high risk and providing evidence for targeted interventions and support. University administrators and supervisors can utilize this scale data to better understand doctoral students’ psychological needs and optimize training environments and mentoring approaches. Furthermore, the scale results can provide empirical foundations for optimizing doctoral education systems, improving academic evaluation mechanisms, and promoting the construction of healthier and more sustainable academic environments.

## 2 Study 1: qualitative exploration

As doctoral students’ time anxiety represents a relatively new research domain, it lacks mature theoretical frameworks and clear conceptual definitions. Study 1 aims to employ grounded theory methodology, starting from doctoral students’ authentic experiences to explore the structural dimensions of doctoral students’ time anxiety, thereby establishing a conceptual foundation for subsequent scale development.

### 2.1 Methods

#### 2.1.1 Participants and sampling strategy

This phase employed a combined strategy of purposive sampling and snowball sampling to select research participants. Initially, purposive sampling was used to precisely identify doctoral students who could provide in-depth information relevant to the research questions, utilizing a brief pre-screening questionnaire to assess their time anxiety levels across dimensions including time management, time scheduling, and psychological–physiological responses. Subsequently, snowball sampling was employed to expand sample coverage, with initial participants asked to recommend other doctoral students meeting the research criteria, particularly individuals from different regions and disciplines. Additionally, participants were further recruited through social media platforms (WeChat, Zhihu, Xiaohongshu, etc.) to enhance the diversity and representativeness of the study.

The research sample encompassed full-time doctoral students from Beijing, Jiangsu, Zhejiang, Sichuan, Guangdong, Hubei, Yunnan, and other regions in China, covering 12 first-level disciplines including natural sciences, engineering, management, education, and law. Participants included first-year students, those undergoing mid-term examinations, students approaching graduation, and those with extended study periods, providing relatively comprehensive coverage of all stages of doctoral study. Following theoretical sampling principles, sample saturation was determined through continuous assessment of whether new data generated novel concepts or theoretical categories. Ultimately, 43 doctoral students participated in formal interviews, comprising 24 male and 19 female students with an average age of 29 years.

#### 2.1.2 Data collection procedures

Data collection primarily employed semi-structured in-depth interviews, supplemented by participant observation and digital ethnography methods. Interviews were conducted between December 2023 and May 2024, with each participant completing at least one formal interview lasting 30–60 min. Various formats were utilized, including face-to-face and video call interviews, to accommodate different participants’ needs.

The interview guide was designed around open-ended questions focusing on themes such as manifestations, influencing factors, and coping strategies related to doctoral students’ time anxiety, including questions such as “Could you describe when you experienced the most intense time anxiety during your doctoral studies?” and “What are the main challenges you face in time management?” Through pilot interviews with 10 doctoral students and review by three experts in higher education management, the interview guide underwent multiple revisions to ensure its scientific rigor and operability. Informed consent forms were distributed before each interview. During interviews, a neutral stance was maintained while encouraging participants to express their views through specific experiences. Interviews were immediately transcribed and accompanied by analytical memos following each session.

As a member of the doctoral student community, the researcher conducted participant observation through participation in laboratory activities, academic conferences, and other events. Additionally, digital ethnography methods were employed to analyze doctoral students’ temporal experience information on relevant social media platforms as supplementary data. Ultimately, approximately 16 h of audio recordings and 100,000 words of textual content were collected.

#### 2.1.3 Data analysis approach

This study employed the systematic grounded theory approach developed by Corbin and Strauss (1990) for data analysis. This method emphasizes maintaining an open stance during the analytical process, extracting key concepts from raw data through systematic coding procedures, revealing the deeper meanings underlying these concepts, and elucidating their interrelationships. Data collection and analysis proceeded simultaneously in continuous cycles until theoretical saturation was achieved.

Coding, as the core component of grounded theory, aims to progressively construct a comprehensive understanding of the research phenomenon through systematic classification and categorization. To ensure efficiency and systematicity in the coding process, all textual materials were imported into NVivo 12 Plus software for multidimensional analysis. The coding work followed three main phases: Open Coding employed a process of “defining phenomena→delimiting concepts→naming categories” to conceptualize and categorize raw data; Axial Coding followed the paradigm model of “causal conditions→phenomena→context→intervening conditions→action/interaction strategies→consequences” to systematically integrate and establish logical relationships between categories; Selective Coding used the “storyline” approach to extract core categories and construct an overall analytical framework.

To ensure analytical quality and credibility, the following quality control measures were implemented:

*Coding consistency*: Three independent researchers (including two doctoral students with extensive experience in doctoral education research and proficiency in grounded theory methodology) participated in the coding process. At the beginning of the study, coders received training on coding rules and procedures to ensure unified understanding of research content and coding standards. Regular discussion meetings were held throughout the coding process to compare and discuss coding results and reach consensus. The inter-coder reliability coefficient was 0.85, indicating good reliability of the coding results. For coding disagreements, collective discussions were conducted with reference to original interview materials until consensus was reached.

*Theoretical Saturation Testing*: This study followed rigorous criteria for judging theoretical saturation. After completing interviews with 31 doctoral students, the reserved interview materials from 12 additional doctoral students were coded following the same coding steps and principles, revealing no new concepts or categories. To ensure reliability of the results, two additional supplementary interview materials were examined, which also produced no new categories or relationships. When multiple consecutive interviews generated no new concepts or categories and new data merely repeated existing codes and categories, theoretical saturation was determined to have been achieved.

*Triangulation*: To ensure comprehensiveness and reliability of research data, this study employed cross-verification through multiple data sources. In addition to in-depth interview data, supplementary data were collected through participant observation (such as participation in laboratory activities and academic conferences), netnography (analyzing doctoral students’ temporal experiences on relevant social media platforms), and literature analysis. Through mutual corroboration and cross-examination of data from different sources, the consistency and reliability of research findings were verified, further enhancing research validity. After interviews, researchers promptly transcribed recordings into text and provided feedback to interviewees for confirmation, ensuring accurate understanding of interview content.

This study strictly adhered to research ethics standards. Data collection was conducted with participants’ full informed consent, anonymization was employed to ensure data confidentiality and protect participants’ rights and privacy, and the study received approval from the institutional ethics review board.

#### 2.1.4 Open coding: generation of initial concepts

In the open coding phase, interview texts underwent repeated comparative analysis line by line and word by word, without presupposing any theoretical framework, transforming raw materials into initial concepts and categories. Researchers first applied labeling processes to raw interview materials, maintaining the authenticity of participants’ original expressions. For example, when participants stated “I can’t sleep every night, always thinking about what if tomorrow’s tasks can’t be completed,” this was labeled as “sleep disturbances.” Subsequently, related labels were categorized, such as integrating “sleep disturbances,” “loss of appetite,” and “accelerated heartbeat” into the “physiological responses” phenomenon. During the conceptualization stage, “physiological responses” was transformed into academic concepts such as “somatic manifestations of time pressure”. Finally, similar concepts were integrated into initial categories, such as combining “somatic manifestations,” “emotional fluctuations,” and “feelings of tension” into the “affective experience” category.

Through repeated comparison, analysis, and integration, and after eliminating concepts that appeared fewer than three times, this study identified 49 valid concepts and 26 initial categories. These initial categories encompassed multiple dimensions of doctoral students’ time anxiety experiences, with typical categories including “time scarcity perception,” “task accumulation anxiety,” “self-doubt,” “procrastination behavior,” and “time control failure” establishing a solid foundation for subsequent axial coding.

#### 2.1.5 Axial coding: inter-categorical relationships and dimensional extraction

The axial coding phase aimed to establish logical relationships between initial categories, forming higher-level main categories. Researchers reclassified, compared, and analyzed the 26 initial categories to explore the intrinsic connections between them. Through systematic analysis, the research found that doctoral students’ time anxiety phenomena could be summarized into three interrelated yet relatively independent main categories:

*Affective anxiety:* This integrated categories related to immediate and intense emotional experiences arising from doctoral students’ temporal perception processes. It primarily includes manifestations such as time pressure sensation, sense of temporal loss of control, feelings of time resource depletion, and future-related fears. Typical interview expressions such as “Every day when I open my eyes, I feel like time is chasing me” reflect intense time pressure sensations.

*Cognitive anxiety:* This encompassed persistent worries arising from doctoral students’ rational thinking, long-term assessment, and future expectations regarding time-related issues. It primarily manifested as time efficiency anxiety, time scarcity anxiety, future uncertainty anxiety, and opportunity cost anxiety. As one participant reflected, “I calculated the time remaining until graduation and looked at the volume of tasks to complete—there really isn’t enough time”, exemplifying typical time scarcity anxiety.

*Behavioral manifestations:* This integrated categories related to various defensive coping strategies adopted by doctoral students in response to time anxiety. It included behavioral patterns such as self-deprivation, avoidance/procrastination, time compression, and excessive time control. For instance, “In order to squeeze out more time for research, I often skip meals and sleep” reflected self-deprivation behavior.

#### 2.1.6 Selective coding: core category confirmation and theoretical integration

In the selective coding phase, the core category “multidimensional structure of doctoral students’ time anxiety” was established, and the “storyline” method was employed to integrate the three main categories—affective anxiety, cognitive anxiety, and behavioral manifestations—constructing a comprehensive theoretical framework.

Through in-depth analysis of the relationships between the three main categories, the research found that they do not exist in isolation but interact to form a dynamic system. Affective anxiety, as the most direct experiential response, influences individuals’ rational assessment of time-related issues; cognitive anxiety, through persistent worries and evaluations, intensifies the magnitude of emotional experiences; behavioral manifestations serve as external expressions of both affective and cognitive anxiety, while their outcomes provide feedback that influences affective and cognitive states, creating cyclical effects. The theoretical storyline constructed by the research is as follows: Doctoral students face unique temporal experience challenges in specific academic environments and, under the influence of multiple factors, develop time anxiety encompassing three dimensions—affective, cognitive, and behavioral. This multidimensional time anxiety forms a self-reinforcing system through internal cyclical mechanisms, continuously affecting doctoral students’ subjective experiences and objective performance.

Theoretical saturation was achieved when newly collected interview data no longer generated new concepts and categories, and the three-dimensional structure could adequately explain all relevant phenomena.

### 2.2 Results

The grounded theory analysis results indicate that doctoral students’ time anxiety comprises three dimensions: affective anxiety, cognitive anxiety, and behavioral manifestations. The analysis generated 49 valid concepts and 26 initial categories, which were ultimately integrated into three main categories. The specific composition of the three dimensions is shown in Table 1.

**Table 1:** Multidimensional constructs of doctoral students’ time anxiety.

Dimension	Conceptual definition	Specific manifestations
Affective anxiety	Immediate, intense emotional experiences and related physiological responses generated by doctoral students during temporal perception processes	Time pressure sensation, sense of temporal loss of control, feelings of time resource depletion, future-related fears
Cognitive anxiety	A series of persistent worries and concerns arising from doctoral students’ rational thinking, long-term assessment, and future expectations regarding time-related issues	Time efficiency anxiety, time scarcity anxiety, future uncertainty anxiety, opportunity cost anxiety
Behavioral manifestations	Defensive coping strategies adopted by doctoral students in response to time anxiety	Self-deprivation, avoidance/procrastination, time compression, excessive time control

## 3 Study 2: initial scale development and pilot testing

Based on the grounded theory analysis results from Study One, Study Two aims to transform qualitative research findings into measurable scale items, forming an initial measurement tool through systematic scale development procedures and conducting pilot testing for validation.

### 3.1 Initial scale development

#### 3.1.1 Item generation

Considering the complex multidimensional nature and highly contextualized characteristics of doctoral students’ time anxiety, this study employed a mixed strategy for item generation, primarily using inductive methods supplemented by deductive approaches, to ensure that the scale possesses both theoretical foundations and alignment with doctoral students’ authentic experiences.

Inductive item development: Based on the grounded theory analysis results from Study One, a “bottom-up” approach was employed to generate items. Through systematic review of 100,000 words of raw interview materials, keywords, phrases, and concepts related to the three dimensions of affective anxiety, cognitive anxiety, and behavioral manifestations were extracted. These elements were subsequently transformed into measurable items, ensuring that each item could accurately capture specific manifestations of doctoral students’ time anxiety. During the item formulation process, particular attention was paid to maintaining consistency with doctoral students’ everyday language to enhance the scale’s applicability. For example, based on participants’ expressions, items such as “I often feel inexplicable tension and pressure when looking at dates on the calendar” and “I feel that my future is uncertain” were generated, totaling 37 initial items.

Deductive item supplementation: Although no measurement tools specifically targeting doctoral students’ time anxiety currently exist, existing related scales provided valuable references for this study. Through systematic review of established scales in related fields such as time anxiety, time pressure, and time perception, including Usunier and Valette-Florence (1994) time style scale, Zaleski (1996) future time anxiety scale, and Liu et al. (2023) teacher time poverty scale, among other classic measurement instruments, 17 items related to doctoral students’ time anxiety were carefully selected and adapted from these scales. Following language adjustment and contextual adaptation, these items were incorporated into the initial item pool.

Through the combination of inductive and deductive methods, an initial item pool for the doctoral students’ time anxiety scale containing 54 items was constructed. The item distribution was as follows: 16 items for the affective anxiety dimension, 23 items for the cognitive anxiety dimension, and 15 items for the behavioral manifestations dimension. The detailed composition of the initial item pool is provided in the Supplementary material.

#### 3.1.2 Content validity assessment

To ensure that the scale items comprehensively and accurately reflect the construct of doctoral students’ time anxiety, the study invited seven experts from relevant fields to conduct content validity assessment, namely, three psychology professors, two education professors, and two supervisors with extensive experience in doctoral education. All experts possessed over 10 years of research and teaching experience, were familiar with scale development procedures, and had in-depth understanding of doctoral education issues.

The expert evaluation employed standardized procedures, with each expert independently assessing the relevance, clarity, and construct representativeness of the 54 initial items. The evaluation used a 5-point Likert scale (1 = completely irrelevant, 5 = highly relevant), while simultaneously collecting experts’ suggestions for item wording modifications and supplementary opinions on construct coverage completeness.

Content validity was quantitatively analyzed through the content validity ratio (CVR), calculated using the formula: CVR = (ne - N/2)/(N/2), where ne represents the number of experts rating 4 or 5 points, and N represents the total number of experts. This study sets the CVR critical value at 0.43, requiring recognition from at least five experts for item retention. Based on expert evaluation results and qualitative feedback, 9 items with CVR values below the critical threshold were deleted, 15 items’ wording was modified, and 2 new items were added following expert suggestions to enhance the completeness of construct measurement.

Through content validity assessment, a pilot version of the scale containing 47 items was ultimately formed, establishing a solid content foundation for subsequent scale development. The item distribution was as follows: 13 items for the affective anxiety dimension, 19 items for the cognitive anxiety dimension, and 15 items for the behavioral manifestations dimension.

### 3.2 Pilot testing

#### 3.2.1 Pilot test design and implementation

To evaluate the comprehensibility and applicability of items, the study conducted a small-scale pilot test. Using a combination of convenience sampling and snowball sampling methods, 45 doctoral students were recruited, including 25 natural sciences doctoral students and 20 humanities and social sciences doctoral students, comprising 22 male and 23 female students with an average age of 28.6 years.

Participants completed the 47-item pilot version of the scale and provided feedback on item comprehensibility and applicability, while completion time was recorded. Analysis results indicated that no items exhibited obvious comprehension ambiguity, demonstrating the scale’s good content appropriateness and semantic clarity. The average response time was approximately 18 min, which was relatively lengthy; however, considering that the primary purpose of this stage was to ensure item quality, the research team decided to retain all items for subsequent large-sample statistical analysis.

#### 3.2.2 Item analysis and selection

Building upon the pilot test, the first round of formal survey was conducted in July 2024 for item analysis. The questionnaire employed a 5-point Likert scale (1 = strongly disagree, 5 = strongly agree) and was distributed to doctoral students through both online and offline channels using convenience and snowball sampling methods. A total of 210 questionnaires were distributed, with 179 valid questionnaires recovered, yielding an effective response rate of 85.24%. The sample distribution is shown in Table 2.

**Table 2:** Sample distribution (first round).

Variable	Category	Sample size	Percentage	Variable	Category	Sample size	Percentage
Demographics	Male	124	69.27%	Institution type	Double first-class universities	122	68.16%
	Female	55	30.73%		Regular universities	57	31.84%
Year	First year	64	35.75%	Admission method	Master-PhD program	22	12.29%
	Second year	50	27.93%		Application assessment	85	47.49%
	Third year	36	20.11%		Regular admission	72	40.22%
	Fourth year	19	10.61%	Discipline	Natural sciences	77	43.02%
	Fifth year and above	10	5.59%		Social sciences	102	56.98%

To validate the content validity of the doctoral students’ time anxiety scale and identify items with good measurement properties, the study employed the critical ratio analysis method to screen scale items based on the pilot test. The sample was ranked by total scores, with the top 27% selected as the high-score group and the bottom 27% as the low-score group, calculating the critical ratio (CR) for each item. Using *p* < 0.05 as the significance criterion, items with good discriminatory power were retained.

Analysis results revealed that among the 13 items in the affective anxiety dimension, 11 items had significant CR values (range: 3.619–17.616), with 2 non-significant items deleted; among the 19 items in the cognitive anxiety dimension, 16 items had significant CR values (range: 2.721–11.339), with 3 non-significant items deleted; among the 15 items in the behavioral manifestations dimension, 11 items had significant CR values (range: 6.026–19.729), with 4 non-significant items deleted (Table 3).

**Table 3:** Summary of item analysis results.

Dimension	Initial items	Significant CR items	CR value range	Deleted items	Retained items
Affective anxiety	13	11	3.619–17.616	2	11
Cognitive anxiety	19	16	2.721–11.339	3	16
Behavioral manifestations	15	11	6.026–19.729	4	11
Total	47	38	2.721–19.729	9	38

Through item analysis, 38 items with good discriminatory power were retained from the 47 initial items, effectively covering the three dimensions of doctoral students’ time anxiety and establishing a foundation for subsequent exploratory factor analysis.

## 4 Study 3: formal scale development and validation

Based on the completion of initial scale development and pilot testing in Study Two, Study Three aims to develop a doctoral students’ time anxiety scale with good reliability and validity through further empirical investigation, employing statistical methods such as exploratory factor analysis and confirmatory factor analysis.

### 4.1 Formal scale development

This phase was based on the 179 valid questionnaire data collected from the first round of survey in Study Two, conducting reliability analysis and exploratory factor analysis on the 38 items retained through item analysis to form the formal scale.

#### 4.1.1 Reliability analysis

Prior to conducting exploratory factor analysis, reliability analysis was first performed on the retained 38 items to further optimize the scale structure and ensure internal consistency and stability of items. The analysis was conducted using SPSS 26.0 software, with Cronbach’s *α* coefficient used to assess internal consistency and corrected item-total correlation (CITC) > 0.4 as the criterion for item retention.

Analysis results: Cronbach’s *α* coefficients for each dimension exceeded 0.7, indicating good internal consistency. Specifically, the affective anxiety dimension had *α* = 0.717, the cognitive anxiety dimension had *α* = 0.762, and the behavioral manifestations dimension had *α* = 0.771.

Item screening: Based on the deletion criterion of CITC < 0.4, a total of 23 items were removed. The affective anxiety dimension deleted 7 items from 16 items (such as A3, A4, A5, etc.), retaining 9 items; the cognitive anxiety dimension deleted 11 items from 13 items (such as B15, B16, B18, etc.), retaining 2 items; the behavioral manifestations dimension deleted 5 items from 9 items (such as C34, C36, C41, etc.), retaining 4 items. Ultimately, 15 items with CITC values all >0.4 were retained.

#### 4.1.2 Exploratory factor analysis

To explore the structural validity of the doctoral students’ time anxiety scale and identify items that do not conform to the expected structure, exploratory factor analysis was conducted on the 15 items retained after reliability analysis to further optimize the scale structure. The analysis included three key steps: data suitability testing, factor extraction, and factor rotation.

Data suitability testing: The KMO and Bartlett’s test of sphericity were used to confirm whether the data were suitable for factor analysis. The results showed a KMO value of 0.812 (above the empirical standard of 0.70), and Bartlett’s test of sphericity yielded a *χ*^2^ value of 1420.248 (df = 105, *p* < 0.001), reaching significance level, indicating the existence of common factors among constructs and suitability for exploratory factor analysis (Table 4).

**Table 4:** KMO and Bartlett’s test.

Index	Statistic	Value
KMO value		0.812
Bartlett’s test of sphericity	*χ* ^2^	1420.248
	df	105
	*p*	0.000

Factor extraction: Principal component analysis was employed, with common factors extracted based on the Kaiser criterion (eigenvalues >1). The results showed that three common factors were extracted, with a cumulative variance explanation rate of 62.788% (Factor 1: 24.120%, Factor 2: 21.124%, and Factor 3: 17.544%), exceeding the acceptable threshold of 50%, indicating that the extracted factors can effectively explain most of the variance in the data (Table 5).

**Table 5:** Total variance explained.

Factor	Initial eigenvalues			Extraction sums of squared loadings			Rotation sums of squared loadings		
	Eigenvalue	Variance explained %	Cumulative %	Eigenvalue	Variance explained %	Cumulative %	Eigenvalue	Variance explained %	Cumulative %
1	3.713	24.755	24.755	3.713	24.755	24.755	3.618	24.120	24.120
2	3.139	20.926	45.682	3.139	20.926	45.682	3.169	21.124	45.244
3	2.566	17.106	62.788	2.566	17.106	62.788	2.632	17.544	62.788
4	0.964	6.428	69.216						
5	0.764	5.093	74.309						
6	0.738	4.920	79.229						
7	0.614	4.096	83.325						
8	0.562	3.744	87.069						
9	0.487	3.249	90.318						
10	0.430	2.869	93.186						
11	0.390	2.600	95.786						
12	0.207	1.383	97.169						
13	0.163	1.084	98.254						
14	0.147	0.982	99.235						
15	0.115	0.765	100.000						

*Factor rotation*: Varimax rotation was employed for orthogonal rotation to obtain a simple and clear factor structure. The rotated factor loading matrix showed that the correspondence between items and factors was highly consistent with theoretical expectations. However, three items (B14, C38, and C40) had communalities below 0.4 and were therefore deleted (Table 6).

**Table 6:** Rotated factor loading matrix.

Item	Factor loadings			Communality (common factor variance)
	Factor 1	Factor 2	Factor 3	
A2	**0.930**	0.029	0.021	0.866
A6	**0.949**	−0.039	0.052	0.906
A8	**0.920**	0.015	0.025	0.848
A11	**0.931**	−0.002	0.047	0.869
B14	0.067	−0.034	**0.597**	0.362
B17	−0.053	0.080	**0.757**	0.583
B19	0.018	−0.001	**0.752**	0.566
B26	0.091	0.090	**0.736**	0.558
B30	0.006	−0.098	**0.755**	0.579
C33	0.052	**0.904**	0.097	0.830
C37	0.060	**0.780**	0.029	0.613
C38	−0.160	**0.530**	−0.008	0.306
C40	−0.230	**0.440**	−0.068	0.251
C42	0.148	**0.773**	0.048	0.622
C44	0.123	**0.802**	−0.053	0.661

Rotation method: varimax.

Bold values indicate the highest factor loading for each item, reflecting the item’s primary factor assignment.

Through exploratory factor analysis, this study obtained a three-dimensional doctoral students’ time anxiety scale, with a dimensional structure consistent with the grounded theory analysis results. Following data optimization, a formal scale was ultimately formed containing three dimensions—affective anxiety, cognitive anxiety, and behavioral manifestations—with each dimension containing four items, totaling 12 items. The analysis results not only validated the structural validity of the scale but also provided a concise and comprehensive tool for measuring doctoral students’ time anxiety, establishing a foundation for subsequent confirmatory factor analysis.

### 4.2 Scale validation

#### 4.2.1 Participants and data collection

To further validate the stability and applicability of the dimensional structure obtained through exploratory factor analysis, a second round of questionnaire survey was conducted. The new round of survey employed the 12-item scale optimized through exploratory factor analysis, distributing questionnaires to doctoral students through both online and offline methods to collect data. A total of 160 questionnaires were collected. After data cleaning, 24 invalid questionnaires with incomplete responses or failing to meet research requirements were excluded, ultimately obtaining 136 valid questionnaires for confirmatory analysis (sample distribution is shown in Table 7).

**Table 7:** Sample distribution (second round).

Variable	Category	Sample size	Percentage	Variable	Category	Sample size	Percentage
Demographics	Male	76	55.88%	Institution type	Double first-class universities	90	66.18%
	Female	60	44.12%		Regular universities	46	33.82%
Year	First year	41	30.15%	Admission method	Master-PhD program	13	9.56%
	Second year	36	26.47%		Application assessment	74	54.41%
	Third year	31	22.79%		Regular admission	49	36.03%
	Fourth year	19	13.97%	Discipline	Natural sciences	57	41.91%
	Fifth year and above	9	6.62%		Social sciences	79	58.09%

#### 4.2.2 Confirmatory factor analysis

AMOS 26.0 was employed to conduct confirmatory factor analysis on the 12-item three-dimensional model, examining the structural validity of the scale from three perspectives: convergent validity, discriminant validity, and model fit.

Convergent validity testing: The results showed that the standardized factor loadings of all dimensional items were >0.7, indicating strong correspondence between items and their respective factors. The four items in the affective anxiety dimension had factor loadings ranging from 0.887 to 0.919, with average variance extracted (AVE) of 0.811 and composite reliability (CR) of 0.945; the four items in the cognitive anxiety dimension had factor loadings ranging from 0.732 to 0.801, with AVE of 0.589 and CR of 0.851; the four items in the behavioral manifestations dimension had factor loadings ranging from 0.770 to 0.870, with AVE of 0.668 and CR of 0.889. All dimensions had AVE values greater than the critical standard of 0.5, and CR values >0.8, far exceeding the acceptable standard of 0.7, indicating that the scale possesses good convergent validity (Table 8).

**Table 8:** Convergent validity test results of confirmatory factor analysis.

Dimension	Item	Factor loading	AVE	CR
Affective anxiety	I often feel that the deadlines for academic tasks are imminent	0.893	0.811	0.954
	I often feel tense and helpless because I cannot independently arrange my time	0.919		
	I often feel guilty and regretful for not making full use of time	0.887		
	When thinking about future academic and career development, I feel anxious and uneasy	0.904		
	I often worry that my learning efficiency is not high enough and I cannot achieve expected goals	0.801	0.589	0.851
Cognitive anxiety	No matter how hard I try, there never seems to be enough time to complete all tasks	0.762		
	I often worry that my growth progress cannot keep up with future developments and changes	0.773		
	I often think about whether I have invested time in the most valuable things	0.732		
Behavioral manifestations	To free up more time, I often choose to sacrifice and compress my life	0.870	0.668	0.889
	I often postpone heavy or difficult tasks until the last moment	0.770		
	I arrange several activities tightly because I do not want to waste time	0.800		
	I have clear requirements for my time arrangement and find it difficult to accept unplanned events	0.825		

Discriminant validity testing: The Fornell–Larcker criterion was employed for testing, where a dimension demonstrates good discriminant validity when its square root of AVE is greater than its correlation coefficients with other dimensions (Henseler et al., 2015). Analysis results showed that the square roots of AVE for the three dimensions (affective anxiety 0.901, cognitive anxiety 0.767, and behavioral manifestations 0.817) were all greater than the inter-dimensional correlation coefficients (affective anxiety and cognitive anxiety r = 0.270, affective anxiety and behavioral manifestations r = 0.520, cognitive anxiety and behavioral manifestations r = 0.295), indicating that the three dimensions possess sufficient distinctiveness and can effectively measure their respective unique theoretical constructs (Table 9).

**Table 9:** Discriminant validity test results of confirmatory factor analysis.

Dimension	Affective anxiety	Cognitive anxiety	Behavioral manifestations
Affective anxiety	**0.901**		
Cognitive anxiety	0.270	**0.767**	
Behavioral manifestations	0.520	0.295	**0.817**

Diagonal values are square roots of AVE.

Bold values on the diagonal represent the square roots of AVE for each dimension.

Model fit testing: Model fit indices comprehensively achieved ideal levels. Absolute fit parameters: *χ*^2^/df = 1.436 (<3), GFI = 0.923 (>0.9), AGFI = 0.883 (approaching 0.9), RMSEA = 0.057 (<0.08); incremental fit parameters: NFI = 0.937 (>0.9), IFI = 0.980 (>0.9), CFI = 0.980 (>0.9), RFI = 0.918 (>0.9); parsimonious fit parameters: PGFI = 0.604 (>0.5). Except for AGFI being slightly below the 0.9 standard, all other indices met or exceeded acceptable standards, indicating good fit between the theoretical model and observed data, validating the rationality of the scale’s three-dimensional structure (Table 10).

**Table 10:** Model fit indices of confirmatory factor analysis.

Index category	Index name	Fit criteria	Test results	Acceptable
Absolute fit parameters	GFI	>0.9	0.923	Accept
	AGFI	>0.9	0.883	Accept
	RMSEA	<0.08	0.057	Accept
Incremental fit parameters	NFI	>0.9	0.937	Accept
	IFI	>0.9	0.980	Accept
	CFI	>0.9	0.980	Accept
	RFI	>0.9	0.918	Accept
Parsimonious fit parameters	CMIN/df	<3	1.436	Accept
	PGFI	>0.5	0.604	Accept

### 4.3 Results

Through systematic reliability analysis, exploratory factor analysis, and confirmatory factor analysis, a doctoral students’ time anxiety scale comprising three dimensions—affective anxiety, cognitive anxiety, and behavioral manifestations—with a total of 12 items was established (see Table 11).

**Table 11:** Final time anxiety scale for doctoral students.

Dimension	Items
Affective anxiety	1. I often feel that the deadlines for academic tasks are imminent
	2. I often feel tense and helpless because I cannot independently arrange my time
	3. I often feel guilty and regretful for not making full use of time
	4. When thinking about future academic and career development, I feel anxious and uneasy
Cognitive anxiety	5. I often worry that my learning efficiency is not high enough and I cannot achieve expected goals
	6. No matter how hard I try, there never seems to be enough time to complete all tasks
	7. I often worry that my growth progress cannot keep up with future developments and changes
	8. I often think about whether I have invested time in the most valuable things
Behavioral manifestations	9. To free up more time, I often choose to sacrifice and compress my life
	10. I often postpone heavy or difficult tasks until the last moment
	11. I arrange several activities tightly because I do not want to waste time
	12. I have clear requirements for my time arrangement and find it difficult to accept unplanned events

Scale structure: The affective anxiety dimension (four items) measures the immediate emotional experiences of doctoral students during temporal perception processes; the cognitive anxiety dimension (four items) measures doctoral students’ rational thinking and persistent worries regarding time-related issues; the behavioral manifestations dimension (four items) measures the defensive coping strategies adopted by doctoral students in response to time anxiety.

Psychometric properties: The scale demonstrated good reliability and validity in this validation sample (*n =* 136). The overall Cronbach’s *α* coefficient was 0.891, with dimensional *α* coefficients ranging from 0.851 to 0.954. Confirmatory factor analysis showed satisfactory convergent validity (AVE values: 0.589–0.811), discriminant validity, and model fit indices. To further validate the scale’s structural stability and applicability across broader populations, Study 4 conducted an independent validation with a large sample (*n =* 695).

Application guidelines: The scale employs a 5-point Likert scoring system (1 = strongly disagree, 5 = strongly agree), with higher scores indicating higher levels of time anxiety.

## 5 Study 4: large-sample independent validation

To further enhance the reliability of research findings and validate the scale’s applicability across broader populations, this study conducted an independent validation with a large sample. By replicating exploratory and confirmatory factor analyses in the new sample, we examined the replicability of the three-dimensional structure and psychometric properties.

### 5.1 Methods

#### 5.1.1 Participants and data collection

Data collection was conducted from August to October 2024. A combination of online and offline methods was employed. Online surveys were distributed through social media platforms including WeChat, QQ, Xiaohongshu, and Douban groups, while offline surveys were distributed using snowball sampling methods. A total of 812 questionnaires were collected during the survey period, and after screening, 695 valid questionnaires were obtained, yielding an effective response rate of 85.59%. The sample demographic characteristics are shown in Table 12.

**Table 12:** Demographic characteristics of the sample (*N =* 695).

Variable	Category	Sample size	Percentage	Variable	Category	Sample size	Percentage
Demographics	Male	366	52.66%	Institution type	Double first-class universities	494	71.08%
	Female	329	47.34%		Regular universities	201	28.92%
Year	First year	242	34.82%	Admission method	Master-PhD program	89	12.81%
	Second year	184	26.47%		Application assessment	318	45.76%
	Third year	110	15.83%		Regular admission	288	41.43%
	Fourth year	91	13.09%	Discipline	Natural sciences	373	53.67%
	Fifth year and above	68	9.78%		Social sciences	322	46.33%

#### 5.1.2 Measures and data analysis

The study employed the 12-item doctoral students’ time anxiety scale established in Study 3, comprising three dimensions: affective anxiety (four items), cognitive anxiety (four items), and behavioral manifestations (four items). All items used a 5-point Likert scale (1 = strongly disagree, 5 = strongly agree), with higher scores indicating higher levels of time anxiety. SPSS 26.0 was used for reliability analysis and exploratory factor analysis, while AMOS 26.0 was used for confirmatory factor analysis.

### 5.2 Results

Reliability analysis showed that the overall Cronbach’s *α* coefficient of the doctoral students’ time anxiety scale was 0.790, meeting acceptable standards and essentially consistent with the results from Study 3 (*α* = 0.891), indicating stable internal consistency of the scale.

Exploratory factor analysis showed a KMO value of 0.826 and significant Bartlett’s test of sphericity (*p* < 0.05), indicating that the data were suitable for factor analysis. Three factors were extracted with a cumulative variance explained of 61.423%. The rotated factor structure clearly demonstrated the three dimensions, with all items loading above 0.65 on their corresponding dimensions, highly consistent with theoretical expectations and previous research findings (see Table 13).

**Table 13:** Results of exploratory factor analysis for time anxiety.

Item	Factor loadings		
	Factor 1	Factor 2	Factor 3
Affective anxiety 1		0.763	
Affective anxiety 2		0.788	
Affective anxiety 3		0.798	
Affective anxiety 4		0.739	
Cognitive anxiety 5	0.790		
Cognitive anxiety 6	0.753		
Cognitive anxiety 7	0.784		
Cognitive anxiety 8	0.775		
Behavioral manifestation 9			0.783
Behavioral manifestation 10			0.763
Behavioral manifestation 11			0.762
Behavioral manifestation 12			0.757
Cumulative variance explained	61.423%		

KMO = 0.826, *P*(Bartlett test) < 0.05.

Confirmatory factor analysis results (see Table 14) showed that the standardized factor loadings of all 12 measurement items exceeded 0.65. The AVE values for the three dimensions were 0.485, 0.494, and 0.470 respectively, approaching or slightly below the recommended threshold of 0.5. However, the composite reliability (CR) values all exceeded 0.7 (0.790, 0.796, and 0.780 respectively), indicating that the scale possesses acceptable convergent validity.

**Table 14:** Results of confirmatory factor analysis for time anxiety.

Scale	Dimension	Factor loading	AVE	CR
Time anxiety	Affective anxiety	0.692	0.485	0.790
		0.719		
		0.703		
		0.670		
	Cognitive anxiety	0.702	0.494	0.796
		0.709		
		0.720		
		0.679		
	Behavioral manifestation	0.698	0.470	0.780
		0.668		
		0.719		
		0.656		

### 5.3 Summary

The large-sample independent validation demonstrated that the doctoral students’ time anxiety scale exhibited good psychometric properties in the new sample. Exploratory factor analysis reconfirmed the three-dimensional structure, confirmatory factor analysis showed satisfactory convergent validity, and reliability coefficients remained consistent with previous studies. The results across three independent samples (Study 2 *n =* 179, Study 3 *n =* 136, and Study 4 *n =* 695, total *n =* 1,010) were highly consistent, robustly confirming the structural stability and replicability of the scale and providing substantial empirical support for the scale’s broad application among doctoral student populations.

## 6 General discussion

### 6.1 Conclusion

This study systematically developed and validated the Doctoral Students’ Time Anxiety Scale using mixed-methods research, yielding the following main conclusions:

First, the three-dimensional structure of doctoral students’ time anxiety was established. Through grounded theory analysis, it was confirmed that doctoral students’ time anxiety comprises three dimensions: affective anxiety, cognitive anxiety, and behavioral manifestations. This provides a clear theoretical framework for understanding doctoral students’ temporal experiences in academic environments. The three dimensions are interrelated and collectively shape doctoral students’ subjective experiences of time while influencing their academic behaviors and mental health status.

Second, the Doctoral Students’ Time Anxiety Scale was successfully developed. Scale development strictly followed international standards, undergoing systematic procedures including item generation, content validity assessment, pilot testing, item analysis, exploratory factor analysis, and confirmatory factor analysis. The final scale contains 12 items measuring three dimensions: affective anxiety (4 items), cognitive anxiety (4 items), and behavioral manifestations (4 items). Through three rounds of independent sample validation (Study 2 *n =* 179, Study 3 *n =* 136, and Study 4 *n =* 695, total *n =* 1,010), the scale demonstrated stable psychometric properties with good reliability, convergent validity, discriminant validity, and model fit.

Third, the effectiveness of mixed methods in scale development was validated. The study employed a sequential mixed-methods approach of “qualitative → quantitative,” combining bottom-up inductive analysis with top-down validation testing, ensuring both ecological validity of theoretical constructs and statistical validity of measurement instruments. The multi-stage independent sample validation design enhanced the robustness of research conclusions, providing an operational paradigm reference for scale development in emerging research areas lacking mature theoretical foundations.

In summary, this scale can serve as an effective tool for assessing the degree of time anxiety among doctoral students, providing a reliable measurement foundation for related research and practical applications.

### 6.2 Research contributions

#### 6.2.1 Theoretical contributions

First, this study expanded the theoretical boundaries of time psychology. This study systematically explored the phenomenon of time anxiety among doctoral students, extending time psychology theory to the field of higher education and enriching the theoretical understanding of temporal experiences in academic environments. The three-dimensional structure not only validates the applicability of classical anxiety theory in new contexts but also provides a new theoretical framework for understanding temporal experiences in academic environments.

Second, this study deepened research on doctoral students’ mental health and clarified the distinctiveness of time anxiety. This study approached doctoral students’ mental health issues from the unique perspective of time anxiety, providing a new conceptual tool for understanding the psychological difficulties faced by this population. Through grounded theory, this study deeply explored the unique connotation of time anxiety, revealing its distinctiveness from general anxiety, depression, or stress.

From a conceptual perspective, time anxiety has a clear temporal orientation, specifically referring to individuals’ perception, evaluation, and coping with temporal passage, time pressure, and temporal value. This differs importantly from related constructs: General anxiety typically refers to broad, uncertain threats and worries lacking a clear focal object, while time anxiety has a specific focus—time itself; depression’s core features are persistent low mood, loss of motivation, and negative cognition, while time anxiety’s core is a sense of temporal urgency and action-driven behavior that may lead to over-activation and compulsive behaviors (such as time compression and excessive planning), which contrasts sharply with depression’s low-motivation state; academic stress emphasizes the imbalance between external academic demands and personal resources, focusing on difficulties in “what to do” while time anxiety reflects individuals’ subjective perception of time, focusing on “when to do it” and “how much time is available”. The three-dimensional structure (affective anxiety, cognitive anxiety, and behavioral manifestations) identified in this study integrates emotional reactions to temporal passage, cognitive evaluation of temporal value, and behavioral strategies for time management, forming a unique theoretical construct focused on the “temporal” dimension.

It must be acknowledged that this study did not simultaneously measure depression, stress, general anxiety, and other related constructs and therefore cannot directly examine the convergent and discriminant validity of time anxiety with these constructs through empirical data—this is an important limitation of this study. Although confirmatory factor analysis showed that the three dimensions of time anxiety are well distinguished from each other, this is limited to within-scale discrimination and has not been compared with external related constructs. Based on existing literature (Levecque et al., 2017; Woolston, 2022), doctoral students indeed face higher mental health risks, and time anxiety may coexist or interact with problems such as depression and stress, but its unique temporal orientation makes it a valuable independent research subject. The definition of this concept and the development of measurement tools provide a foundation for subsequent research to distinguish the relationships and mechanisms between time anxiety and other mental health problems, contributing to the construction of a more comprehensive theoretical system for doctoral students’ mental health.

Third, this study advanced the application of mixed methods in scale development. This study demonstrated a scale development pathway combining grounded theory with psychometrics, providing methodological insights for measurement tool development in psychology and education. This sequential mixed-methods approach of “qualitative → quantitative” effectively balances theoretical depth with measurement precision, and the multi-stage independent sample validation design enhances the robustness of research conclusions, providing an operational methodological framework for similar research.

#### 6.2.2 Practical contributions

First, this study provided a scientifically reliable measurement tool. The Doctoral Students’ Time Anxiety Scale provides higher education administrators, mental health service providers, and researchers with a specialized assessment tool that can be used to identify high-risk individuals, evaluate intervention effects, and conduct large-scale survey research. The development of this scale fills a gap in the measurement of doctoral students’ time anxiety, providing important support for promoting the professionalization of doctoral students’ mental health services.

Second, this study guided doctoral education practice. The three-dimensional structure of time anxiety revealed by this study provides specific guidance for optimizing doctoral education environments. Educational administrators can design targeted support measures from three levels—emotional support, cognitive guidance, and behavioral regulation—to improve doctoral students’ temporal experiences and mental health status and promote the optimization and improvement of doctoral education models.

Third, this study promoted the development of mental health services. The development of the scale provides a specialized tool for doctoral students’ mental health services, helping counselors and therapists more accurately assess doctoral students’ time anxiety status, develop personalized intervention plans, improve the professional level and effectiveness of mental health services, and advance the improvement of mental health service systems.

### 6.3 Limitations and future directions

Although this study has made important progress, certain limitations remain, and future research needs further improvement:

First, sample representativeness needs expansion. Although the research sample covered doctoral students from different regions, disciplines, and grade levels and achieved larger scale and more balanced sample distribution in Study 4, it still mainly focused on full-time doctoral students in China. This study did not include part-time doctoral students and students with disabilities, groups that may face unique temporal pressures and anxiety experiences. Future research needs to validate the scale’s applicability and measurement equivalence in broader cultural contexts and populations, conduct cross-cultural validation studies, explore the impact of cultural factors and learning modes on doctoral students’ time anxiety, and promote the internationalization of this field.

Second, validity testing needs deepening. This study mainly focused on establishing structural validity, but further improvement is needed in the following aspects: (1) Criterion-related validity: Future research should employ more external criteria (such as academic performance, graduation delay rates, mental health indicators, and life satisfaction) to comprehensively validate the scale’s predictive validity and practicality. (2) Convergent and discriminant validity: An important limitation of this study is that it did not simultaneously measure depression, general anxiety, academic stress, and other related mental health constructs and therefore cannot directly examine the relationship between time anxiety and these constructs through empirical data. Although this study established the unique conceptual connotation of time anxiety through theoretical analysis and grounded theory methods, future research still needs to empirically validate the convergent and discriminant validity of time anxiety through multi-scale administration, correlation analysis, incremental validity testing, and other methods, clarifying its unique value in doctoral students’ mental health research. (3) Measurement bias: This study’s use of self-report methods may involve social desirability bias; future research can incorporate behavioral observation, physiological indicators (such as heart rate variability and cortisol levels), time logs, and other multi-method validation. (4) Measurement invariance: Longitudinal studies are needed to explore the dynamic patterns of change in doctoral students’ time anxiety and to improve testing of measurement invariance across gender, discipline, and cultural groups.

Third, theoretical mechanisms and intervention applications need expansion. Based on the scale, further exploration is needed of the generation mechanisms, influencing factors, and consequences of doctoral students’ time anxiety to construct a more complete theoretical model. Meanwhile, targeted intervention programs should be designed and validated based on scale measurement results, promoting the translation of theoretical research into practical applications, and exploring the scale’s applicability in related academic populations (such as master’s students, young faculty, and postdoctoral researchers) to provide scientific support for constructing healthy academic environments.

## Data Availability

The original contributions presented in the study are included in the article/Supplementary material, further inquiries can be directed to the corresponding author.
