# Supplementary material for: Development and validation of the doctoral students’ time anxiety scale
Source: Front Psychol. 2026 May 14;17:1710486. doi: 10.3389/fpsyg.2026.1710486 (PMC13216462; doi:10.3389/fpsyg.2026.1710486)
Supplement: Supplementary file 1 [file Supplementary_file_1.pdf]

Appendices:

Appendices Composition of initial time anxiety scale for doctoral students

Dimension	Manifestation	Item	Source
A. Affective Anxiety Dimension	Time Pressure	A1. When looking at dates on the calendar, I often feel inexplicable tension and pressure	Interview
		A2. I often feel that the deadlines for academic tasks are imminent	Interview
		A3. Even when I have sufficient time, I often feel time pressure and cannot relax	Expert
	Time Loss of Control	A4. I feel that I have less and less control over time, always being pushed by other affairs	Interview
		A5. Faced with multitasking and frequent interruptions, I feel unable to effectively manage my time	Interview
		A6. I often feel tense and helpless because I cannot independently arrange my time	Interview
		A7. I have no autonomy in my time allocation	Liu et al.
	Time Resource Loss	A8. I often feel guilty and regretful for not making full use of time	Interview
		A9. Looking back, I often worry that I wasted too much time on unimportant things	Interview
		A10. Recalling wasted study time, I always feel deep regret and self-blame	Interview
	Future Fear	A11. When thinking about future academic and career development, I feel anxious and uneasy	Interview
		A12. When I think about a future day, month, or year, I tremble with fear	Zaleski
		A13. I worry that I will be unable to overcome increasing difficulties	
B. Cognitive Anxiety Dimension	Time Efficiency Anxiety	B14. I often doubt whether I have effectively utilized my study and research time	Interview
		B15. I feel that my time is fragmented into many pieces, making it difficult to concentrate on completing one task	Interview

		B16. I often feel that I haven’t fully realized the value of time, which makes me very anxious	Interview
		B17. I often worry that my learning efficiency is not high enough and I cannot achieve expected goals	Interview
		B18. I sometimes feel that the way I use time is useless and worthless	Usunier
	Time Scarcity	B19. No matter how hard I try, there never seems to be enough time to complete all tasks	Interview
		B20. I often feel that time is seriously insufficient, making it difficult to balance study, research, and personal life	Interview
		B21. I often feel that time passes particularly fast and worry about being unable to complete set goals	Interview
		B22. I often worry about another day passing without finishing work	Interview
		B23. I feel that time is not enough	Rudd
	Future Uncertainty Anxiety	B24. Faced with future uncertainties, I find it difficult to make long-term time planning and arrangements	Interview
		B25. I feel uncertain about my academic and career prospects, which makes me very anxious	Interview
		B26. I often worry that my growth progress cannot keep up with future developments and changes	Expert
		B27. I feel that my future is uncertain	Interview
		B28. I spend time thinking about what my future might look like	Usunier
	Opportunity Cost	B29. When I am doing one thing, I worry about missing more important other things	Interview
		B30. I often think about whether I have invested time in the most valuable things	Interview
	Anxiety	B31. I often hesitate between different choices, worrying about making wrong time allocations	Interview
		B32. Faced with the current situation, I often doubt whether my decision to pursue a PhD is correct	Interview
C. Behavioral Manifestations Dimension	Self-Deprivation	C33. To free up more time, I often choose to sacrifice and compress my life	Interview
		C34. Even on rest days, I feel guilty for not studying	Interview

	C35. I often feel like a machine, working constantly without time to relax	Interview
	C36. To complete academic tasks, I often sacrifice sleep and entertainment time	Interview
Avoidance/Procrastination	C37. I often postpone heavy or difficult tasks until the last moment	Interview
	C38. When faced with heavy or difficult tasks, I sometimes choose to temporarily avoid them	Expert
	C39. I sometimes put off what I should do today until tomorrow	Interview
Time Compression	C40. Always trying to complete tasks in the shortest time, even if it might affect quality	Interview
	C41. To save time, I often quickly browse literature rather than read it in depth	Interview
	C42. I arrange several activities tightly because I don't want to waste time	Expert
	C43. Even when there's nothing urgent, I walk fast	
Excessive Time Control	C44. I have clear requirements for my time arrangement and find it difficult to accept unplanned events	Interview
	C45. I am very afraid that some unexpected events will disrupt my original planned arrangements	Expert
	C46. I like to follow schedules	Usunier
	C47. If tasks keep changing, it gives me a sense of urgency	Chong
